# Effect of pH on lipid oxidation of red onion skin extracts treated with washed tilapia (Oreochromis niloticus) muscle model systems

**DOI:** 10.3906/kim-2004-47

**Published:** 2020-12-16

**Authors:** Senem GÜNER, Yavuz YAĞIZ, Zeynal TOPALCENGİZ, Hordur G. KRISTINSSON, George BAKER, Paul SARNOSKI, Bruce A. WELT, Amarat SIMONNE, Maurice R. MARSHALL

**Affiliations:** 1 Food Science and Human Nutrition Department, University of Florida, Gainesville, FL USA; 2 Department of Food Engineering, Faculty of Engineering and Architecture, Muş Alparslan University,Muş Turkey; 3 Matis ohf., Vinlandsleid, Reykjavik Iceland; 4 Department of Agricultural and Biological Engineering, Institute of Food and Agricultural Sciences, University of Florida, Gainesville, FL USA; 5 Institute of Food and Agricultural Sciences, University of Florida, Gainesville, FL USA

**Keywords:** Onion skin, polyphenols, antioxidants, lipid oxidation, washed muscle system, waste management

## Abstract

The aim of the study was to investigate the effect of pH on the lipid oxidation of red onion skin extracts (ROSEs) treated with washed tilapia muscle model systems (WTMS). Minced and buffered washed samples were prepared at pH 6.3 and 6.8. The WTMS were treated with2 different concentrations of red onion skin prior to storage for 5 days. Lipid oxidation was investigated via peroxide values (PVs), thiobarbituric acid reactive substances (TBARS), and the formation of volatile compounds. Fatty acid profiles of the samples were also identified. The ROSEs were able to significantly suppress the PV (~71%) and TBARS (~42%) formation. Hexanal and octanal formations in the WTMS were relatively less in the ROSE-treated samples. The WTMS samples prepared at pH 6.3 were more vulnerable to lipid oxidation than those prepared at pH 6.8. Red onion skin polyphenols may increase the lag phase of lipid oxidation, depending on pH levels, resulting in the shelf life extension of raw fish.

## 1. Introduction

The color and odor of fish meat are 2 of the most important factors affectingproduct quality and consumer demands. Fish isprone to lipid oxidation during long periods of storage under unsuitable conditions, as the result of temperature abuse, due to the high unsaturated fatty acid (FA) content [1]. The common undesired outcomes of lipid oxidation are the production of off-flavors and odors, discoloration, and loss of nutrients [2,3]. The formation of volatile lipid byproducts and free FAs are another quality factor affecting the sensory properties of fish meat [1]. Hydroperoxides formed through lipid autooxidation can easily turn into some off-flavor compounds, including volatile compounds, such as aldehydes, ketones, and alcohols[3,4].

The presence of antioxidant and prooxidant compounds, such as polyphenols, with subgroups including flavonoids and anthocyanins, can reduce the effect of lipid oxidation [5–7]. Onions contain high amounts of polyphenols, including quercetin aglycone, and glycosides as the main flavonoids [8]. The stability of anthocyanin compounds is highly dependent on factors such as oxygen, temperature, metallic ions, pH, and copigments [9]. Onions are known as a good abundant source of secondary metabolites, such as flavonoids. In addition to their bioactive attributes, flavonoids can also increase the antioxidant capacity of present anthocyanins, and quercetin is one of the appropriate copigments naturally found in onions [8,10]. The antioxidant activity, resonance, and hydrogen bonding of anthocyanins are strongly affected by pH [11]. Fish hemoglobin is a lipid oxidation initiator and oxidation usually increases when the pH level decreases [12]. The effect of pH on antioxidant activity used in a food model may change the stability of many bioactive compounds found in polyphenol-rich sources.

When investigating the effects of possible antioxidants on lipid oxidation in seafoods, the results can be overestimated in the presence of interior antioxidants and prooxidants. A model system of washed meat muscle can be used in order to overcome the consistency problem [13–16]. The removal of antioxidants and prooxidants from fish meat samples provides more reliable investigations regarding the outcomes of lipid oxidation with some determined external factors. Small amounts of prooxidants can pass onto foods from water, ingredients, or equipment used in production and adversely affect oxidation [17]. The meat muscle samples are washed in order to remove the internal antioxidants and leave the high degree of polyunsaturated FAs. These FAs enable an aimed initiation of lipid oxidation in a convenient time period. Thus, iron and hemoglobin can be eliminated to avoid undesired interference [14,15]. The remaining muscle part generally contains myofibrillar proteins and membrane phospholipids. Additionally, since pH levels can directly influence some physiochemical actions, including lipid oxidation, applications of different pH levels may aid in observing the effectiveness of such antioxidant compounds, such as polyphenols, on lipids.

Lipid oxidation can be reduced by conventional methods, such as cooling, freezing, and storing on ice. Moreover, synthetic antioxidants can be added to products [2,3,18]. Sources of natural antioxidants are commonly preferred by producers to reduce costs and eliminate consumer concerns about their synthetic counterparts. Polyphenols are found abundantly in onion skin, a main waste for the food industry, and can substitute synthetic antioxidants in order to delay/prevent lipid oxidation occurring in muscle foods. To reduce the adverse effects of lipid oxidation in seafoods, polyphenols extracted from onion skins can be used as natural lipid oxidation inhibitors. The aim of this study was to investigate the effects of the polyphenols in onion skins on lipid oxidation in a washed tilapia ( *Oreochromis niloticus* ) meat model at different pH levels.

## 2. Materials and methods

### 3 and 68 2.1. Preparation of the washed tilapia muscle model system at pH 6.3 and 6.8

The washed tilapia muscle model system (WTMS) was prepared following the method described by Halldorsdottir et al. [19], with some modifications. Briefly, fresh and farm-raised tilapia samples were bought from a seafood retail establishment in Gainesville, Florida, USA. Within a couple of hours, the fish were beheaded, skinned, and filleted. Inedible muscle and fat tissues on the fillet were also removed. The remains were thoroughly homogenized using a sterile STX Turbo Force food grinder (STX International, Lincoln, NE, USA). Samples were kept cool on ice at all times. Minced samples were washed 2 times with deionized water (1:3, w/w), followed by washing with 50 mM of sodium phosphate buffers. The buffers were prepared at 2 different pH levels, 6.3 and 6.8. These pH levels were determined based on the preliminary results from the laboratory showing the significant effects of polyphenol extracts on lipid oxidation. The ratio of sample to buffer was 1:3 (w/w). WTMS homogenates were then vacuum packaged and stored at –80°C for future use.

### 2.2. Red onion skin extract preparation

Dry red onions ( *Allium cepa* L.) were bought from a store in Gainesville, Florida, USA. The red onion skins were then removed using a sterile knife to the semiwet layer of the onion. The skins were cleaned and sorted, and then the samples were kept in an Advantage freeze dryer (The Virtis Co., Gardiner, NY, USA) for 2 days. Dried samples were then thoroughly pulverized using an Omni-Mixer 17105 grinder (OCI Instruments, Inc., Waterbury, CT, USA) for homogenous consistency. The red onion skin extracts (ROSEs) were prepared at 2 different concentrations in the following manner. First, 2.5 and 5.0 g of powdered skin samples were extracted in an acetone and water solution with acetic acid (70:29.9:0.1, v/v, respectively),as described by Xu et al. [20]. All of the solvent was removed by vaporization under a vacuum using a Büchi rotary evaporator (BÜCHI Labortechnik AG, Flawil, Switzerland). The obtained extract was then mixed with35 mL of deionized water. The ROSEs were added as 0.005% (v/w) to the WTMS based on the preliminary results.

### 2.3. Oxidation initiation and experimental design

To catalyze lipid autooxidation, a mixture of ferrous sulfate and ascorbic acid (FeAA) was made, as described by Mei et al. [21], with slight modifications. In short, lipid oxidation was initiated by an iron-ascorbic acid redox cycle. A ratio of 500:1000 µmole/kg ferrous sulfate and ascorbic acid, respectively, was found to be most effective on lipid oxidation.

The samples of WTMS at different pH levels, 6.3 and 6.8, were grouped as the control (without any initiator or onion extract), ROSE 1 (with the addition of FeAA and low concentration ROSE), ROSE 2 (with FeAA and a high concentration of ROSE), and a FeAA control (with only oxidation initiator). To investigate the effectiveness of the ROSEs on lipid oxidation, the control (untreated with initiator or onion extract) and treatment groups were stored in sterile petri dishes at 4 ± 1 °C for 5 days. One gram of each sample was randomly collected from a bulk and stored in a sterile tube for day 0 and on each of the 5 days of storage.

### 2.4. Peroxide values

The pH values of the control and treated samples were measured on each of the 5 days of storage with a Fisher Scientific Accumet Basic AB15/157 electronic pH meter ( Thermo Fisher Scientific Inc., Pittsburg, PA, USA). The peroxide values (PVs) of the samples were measured following the method described by Yarnpakdee et al. [22], with some modifications. Briefly, 1 gram of samples was homogenized thoroughly with 10 mL of chloroform-methanol (1:1, v/v) solution and vortexed for 30 s. Later, 3 mL of 5% sodium chloride solution was added to the mixtures for centrifugation at 3000 *Xg* for 10 min at 4 °C. The chloroform phase (2 mL of the lower phase) was transferred into another tube and mixed with 8 mL of chloroform:methanol (1:1, v/v). Ammonium thiocyanate (50 µL, 30%) and iron (II) chloride solution (50 µL, mixture of 0.8% barium chloride and 1% ferrous sulfate) were added to the mixtures. They were kept for 5 min and the absorbances of each were recorded at 500 nm. In order to obtain a standard curve, cumene hydroperoxide (CPO) was prepared at different concentrations, ranging from 0 to 20 µmole/L. Results were expressed as µmole CPO/kg.

### 2.5. Thiobarbituric acid reactive substances

The thiobarbituric acid reactive substance (TBARS) values of the samples were measured following the method of Dekkers et al. [23], with slight modification. The samples (1g) were mixed with 5 mL of 7.5% trichloroacetic acid solution. Next,they were filtered through Whatman No. 1 filter paper. The 2 mL of filtrates were mixed with 0.02 M of TBA solution (2 mL). The mixtures were kept in hot water (~100 °C) for 40 min followed by a quick cooling in iced water for 5 min. The absorbance levels were recorded at 532 nm. In order to obtain a standard curve, 1,1,3,3-tetraethoxypropanewas prepared at different concentrations. The TBARS values were expressed as µmole MDA/kg.

### 2.6. Volatile compounds

Three grams of the control and treated samples were kept in individual 40-mL amber glass solid phase microextraction (SPME) vials equipped with a polythetrafluoroethylene septa and screw caps, with added sodium chloride (table salt) (1:1, w/w). Volatile compounds from the samples were collected on days 0, 3, and 5 of storage and analyzed using gas chromatography (GC) with a mass spectrometry (MS) system for compound identification and a GC with a flame ionization detector (FID) for the selected compound quantification. The volatile compounds were collected with a SPME fiber. Prior to sample collection, the SPME fiber was conditioned at 270 °C for 1 h. The conditioned SPME was exposed to the sample in the amber vial at 45 °C for 30 min. After exposure, the fiber was loaded onto the GC injection port and kept for 5 min. Helium was used as carrier gas at a total flow rate of 3.9 mL/min with 235.8 kPa pressure.

GC/MS conditions were prepared following the method of Iglesias & Medina [24], with some modifications. The Shimadzu GC-2010 Plus GC system (Shimadzu Corp., Kyoto, Japan) with a FID and an RTx-5 w/guard capillary column (30 m × 0.25 mm × 0.2 µm) were used for the quantification of volatile compounds. The Shimadzu GCMS-QP2010 SEGC/MS system (Shimadzu Corp.) was used for qualification of the major peaks. In both systems, the injector temperature was held at 250 °C. The column temperature was initially kept at 35 °C for 3 min, increased to 70 °C, at increments of 30 °C/min, increased to 200 °C, at increments of 10 °C/min, and finally increased to 260 °C, at increments of 20 °C/min, and held at this temperature for 5 min.

### 2.7. Fatty acid profile

FA methyl ester (FAME) analysis was conducted to determine the FA profiles of WTMS samples. Lipid extraction was obtained by following the method of Bligh and Dyer [25]. After lipid extraction, the FAME analysis was conducted. For the FAME analysis, the method of the American Oil Chemists’ Society [26] was used, with some modifications. Briefly, 100 µL of fat was mixed with 1 mL of sodium methoxide in methanol solution. The mixture was kept at room temperature for 20 min and vortexed for 5s. Following that, 0.5 mL of isooctane and 0.2 mL of sodium bisulfate were added to the mixture and vortexed. The mixture was centrifuged at 12,000 *Xg* for 3 min. Next, 0.2 mL of the upper layer was combined with 0.2 mL of hexane in an amber GC vial before analysis. GC conditions were set based on the modified method described by Yagizet al. [27] for optimization of the system for the WTMS samples. Briefly, the FAMEs of samples were investigated using an Agilent GC HP 6890 system (J&W Scientific Inc., Santa Clara, CA, USA), equipped with a FID and an Agilent DB 225MS capillary column with a 30 m × 0.25 mm × 0.25 µm film (J&W Scientific Inc.). Split injection with a split ratio of 50:1 was conducted. The injection port and detector temperatures were 230and 275 °C, respectively. The initial oven temperature was 120 °C with a gradual heating to 220 °C at a rate of 4 °C/min, held for 35 min. The total run time of the analysis was 60 min. Helium was used as the carrier gas at a rate of 0.8 mL/min. Compounds were identified by comparing the retention times of the samples with the retention times of known standards (FAME Mix 37, Supelco; Sigma-Aldrich Corp., St. Louis, MO, USA).

### 2.8. Statistical analysis

JMP Pro 11 (SAS Institute Inc., Cary, NC, USA) was used to analyze the obtained data. Analysis of variance (1-way ANOVA) was conducted and the differences in the means were compared using the Tukey studentized range test. The data were prepared as an average of individual duplicates ± standard deviation. Statistical significance was accepted as P ≤ 0.05.

## 3. Results and discussion

### 3.1. Peroxide values

**Figure 1:**
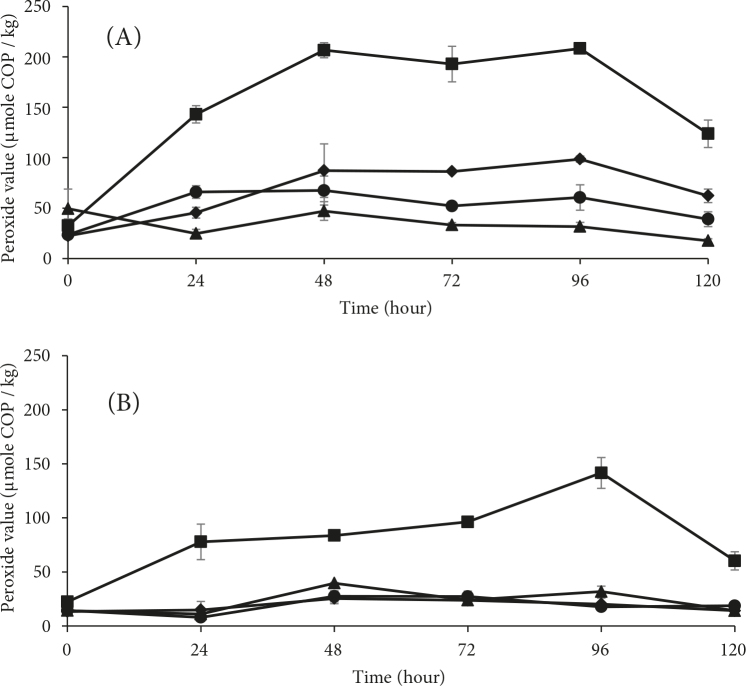
Changes in the PVs of the WTMS samples (▲) without oxidation initiator or ROSEs, (□) with oxidation initiator and FeAA, and (●) ROSE 1 and (♦) ROSE 2 with oxidation initiator and ROSEs at pH (A) 6.3 and (B) 6.8, obtained over 5 days of storage.

All of the WTMS samples at the initial pH values of 6.3 and 6.8 had a stable change in pH between 6.5 and 6.8 under all of the tested conditions during storage, starting from 0 h (data is not shown). Lower pH levels may have increased the initiation step of lipid oxidation, resulting in higher PV formation. Figure 1 shows changes in the PVs of the control and treated samples prepared at pH levels of 6.3 and 6.8 for the 5 days of storage. The effect of pH on the peroxide levels was observed to be stronger for the samples at pH 6.3 in the control groups when compared to those of the samples at 6.8, as was expected. The FeAA groups had the highest peroxide levels of all of the groups when compared to the samples treated with ROSE. The PVs of the control and treated samples at pH 6.3 increased up to day 2, followed by a slow decrease until day 5. Thiansilakul et al. [3] reported that the PVs of tilapia significantly ascended within the first 3 days and later gradually decreased over 15days of iced storage. Maqsood and Benjakul [28] reported that tannic acid (400 ppm) decreased the peroxide levels of hemoglobin-mediated Asian sea bass muscle. For the treated samples at pH 6.8, the PV trends were similar, yet lower, when compared to the values of previous studies. Differencesmay have occurred due to factors such as the useof different muscle systems, oxidation initiators and antioxidants, the pH levels of the samples, and storage temperatures. Moreover, low temperatures, such as iced storage, may increase the lag period of lipid oxidation and primary oxidation products.

Primary lipid oxidation products occur in the presence of unsaturated membrane phospholipids due to their large surface areas, since they can easily come into contact with the lipid oxidation initiators, generally positioned in the aqueous phase of muscle cells[29]. The ROSEs, at both concentrations,were able to successfully delay the oxidation at both prepared pH levels. Samples treated with low ROSE concentrations showed lower products of lipid oxidation than those that were treated with higher concentrations. These results may have occurred because polyphenols can promote membrane structure and function, and protect lipids from the destructive products of oxidation. Additionally, flavanols are very effective in the prevention of lipid oxidation. Their preferential location and orientation in the membrane bilayer can enable their ability of scavenging alkoxyl and hemin radicals [30]. Red onions are known as a rich source of phenolic compounds, particularly quercetin and cyanidin. These compounds candonate a hydrogen molecule to the free radicals. As a result, they become radicals with lower energy and turn into harmless molecules. Phenolic compounds can bind the oxygen atoms of their hydroxyl groups with the free iron and decrease the adverse effects of iron on lipid oxidation [31]. Secondary products of plants are strong antioxidants, yet the attributes of polyphenols, such as chemical structure, aglycone form, and concentration level, can alter their effectiveness on lipid oxidation. For example, Özen and Soyer [32] found that grape seed extract and pomegranate rind extract were able to suppress PV production, whereas green tea extract did not have any inhibition effect.

### 3.2. Thiobarbituric acid reactive substances

**Figure 2:**
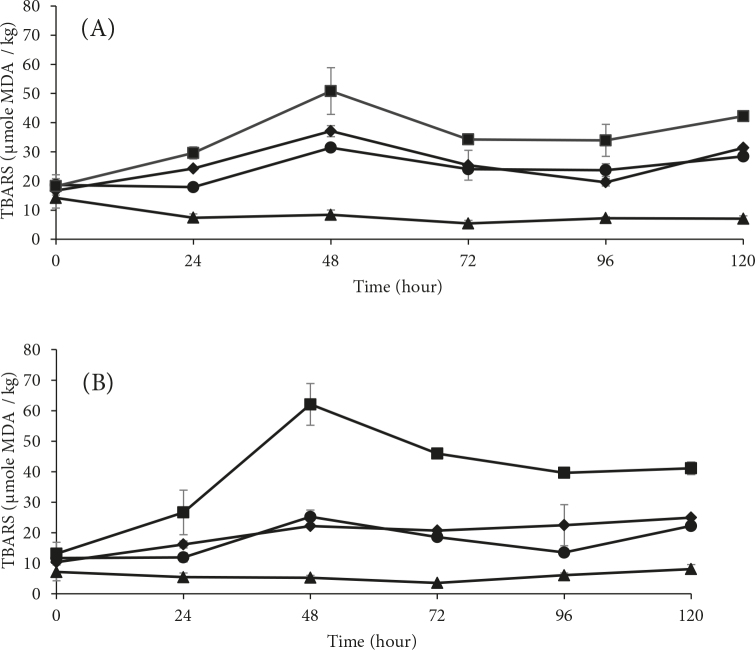
Changes in the TBARS values of the WTMS samples (▲) without oxidation initiator or ROSEs, (□) with oxidation initiator and FeAA, and (●) ROSE 1 and (♦) ROSE 2 with oxidation initiator and ROSEs at pH (A) 6.3 and (B) 6.8, obtained over 5 days of storage.

Figure 2 shows the TBARS levels of the controls and treated samples prepared at pH 6.3 and 6.8 and kept in storage for 5days. TBARS production increasedfor the first 2 days of storage and started to slightly decrease after the third day. The levels of the samples prepared at pH 6.3 and 6.8 were not significantly different (P > 0.05). As expected, the FeAA groups at both pH levels expressed higher TBARS levels than the other treatment groups and control groups (P ≤ 0.05). The control groups at both pH levels did not have significant TBARS production, which was expected due to the absence of the lipid oxidation initiator. The ROSEs were found to be more effective in preventing lipid oxidation from occurring in the samples prepared at pH 6.8. The ROSEs had higher levels of quercetin aglycone (data not shown), enabling more adverse effectiveness on lipid oxidation. Similarly, the extraction of cranberry juice powder, with quercetin aglycone, was found to suppress the lipid oxidation measured as secondary oxidation products in mechanically-separated turkey [18]. In another cranberry study by Lee et al. [33], fractions of cranberry powder, with approximately 74 µmole of quercetin/kg, inhibited almost the 94% of the TBARS formation in a washed cod muscle system at pH 6.3 during storage at 2 °C.

The PV and TBARS levelsin this study were in accordance with those of previous studies. Formation of TBARS may have been limited as a result of the inhibition of the primary oxidation products, which were also observed in the peroxide results. Glycosylation may also have reduced the effectiveness of the quercetin and cyaniding on radical scavenging. In order to achieve suppression of lipid oxidation by phenolic compounds, antioxidant molecules should be located in the lipophilic membraneof the muscle system to inactivate the lipophilic hemin radicals and alkoxyl radicals released from heme-lipid peroxide reactions [33]. As the polyphenol molecule gets bigger, its antioxidant activity on hemoglobin-mediating lipid oxidation can decrease [33]. Thus, the amount of polyphenol used is quite important for its effectiveness. Higher levels may expose prooxidant effects [34]. For example, lipid oxidation measured as TBARS increased at a low pH in a sodium dodecyl sulphate emulsion system, caused by the increased iron stability and/or ascorbate stability [21]. The iron-ascorbic acid complex might have become stronger as the pH of the system decreased.

In this study, the polyphenol was extracted with a solution made of acetone in water with acetic acid, yet polyphenols were applied in deionized water to the WTMS system. The highly extracted polyphenols in the aqueous solution may have attached to the polar ends of the phospholipids. As a result, biomembrane fluidity may have altered, and polyphenols may have chelated ferrous ions before they could enter the membrane. Even though the ROSE groups expressed similar trends as the FeAA groups, following day 1, the lipid oxidation levels dramatically decreased. Phenolic compounds were generally found to be more effective in delaying the induction periods than the formation of TBARS [29], in accordance with this study.

### 3.3. Volatile compounds

Table 1 shows the volatile compound profiles identified in the control and ROSE-treated WTMS samples prepared at 2 different pH levels. In later periods of storage, first, the lipid oxidation products, hydroperoxides, decompose and alcohols, ketones, and aldehydes were produced. Almost all of the samples at pH 6.3 had octen-3-ol and 2,3-octanedione, yet these volatiles were only found in the FeAA groups at pH 6.8 on day 5. This might have been related that other volatiles that occurred, which might have suppressed their peaks due to their low amounts. This phenomenon could also be used to explain the observed loss of some minor compounds and decrease or increase of some compounds found in the samples. Thus, their amounts might have been sufficient to be noticeable in the FeAA samples at pH 6.8. Additionally, the ROSEs might have been strong enough to inhibit the formation of those compounds, as in accordance with the low amounts of TBARS. In order to observe the differences among the control and ROSE-treated groups, major peaks of the volatiles were selected for comparison.

**Table 1:** Volatile compound detection in the WTMS samples prepared at pH 6.3 and 6.8 on days 0, 3, and 5 of storage.

	*Days 0, 3, and 5 at pH 6.3				*Days 0, 3, and 5 at pH 6.8			
Compound	Control	ROSE 1	ROSE 2	FeAA	Control	ROSE 1	ROSE 2	FeAA
Butanol	P, N, N	N, N, N	N, N, N	N, N, N	N, N, N	N, N, N	N, N, N	N, N, N
1-butanol	N, N, P	N, P, N	N, P, N	N, N, N	N, N, P	N, N, P	N, P, P	N, P, P
3-methyl-butanal	N, N, N	N, N, N	P, P, N	N, P, N	N, N, N	N, N, N	N, N, N	N, N, N
1-penten-3-ol	N, N, N	P, N, N	N, N, N	P, N, N	N, N, N	N, N, N	N, N, N	N, N, N
Pentanal	N, N, P	N, N, N	N, N, P	P, N, P	N, N, N	N, P, P	P, P, P	P, P, P
2-methyl-pentanal	N, N, N	P, N, P	N, N, N	N, N, N	N, N, N	N, N, N	N, N, N	N, N, N
1-pentanol	N, P, P	P, P, P	P, P, P	P, P, P	P, P, P	P, P, P	P, P, P	P, P, P
Hexanal	P, P, P	P, P, P	P, P, P	P, P, P	P, P, P	P, P, P	P, P, P	N, P, P
Hexanol	N, N, N	N, N, N	N, N, N	N, N, P	N, N, N	P, N, N	N, N, N	P, P, N
1-hexanol	P, P, N	N, N, N	N, P, N	N, P, N	P, N, P	N, N, N	N, N, N	N, N, N
2-ethyl-hexanol	N, N, N	N, P, N	N, N, N	N, N, N	N, N, N	N, N, N	N, N, N	N, N, N
Heptanal	P, N, P	P, P, P	P, N, P	P, N, P	N, N, N	N, P, P	P, P, P	P, P, P
1-heptanol	N, N, N	N, N, N	N, N, N	N, N, P	N, N, N	N, N, N	N, P, N	P, P, N
1-octen-3-ol	P, N, P	P, P, P	P, P, P	P, N, P	N, N, N	N, N, N	N, N, N	N, N, P
2,3-octanedione	P, N, P	P, N, P	P, N, P	P, N, P	N, N, N	N, N, N	N, N, N	N, N, P
Octanal	P, P, P	P, N, P	P, N, P	P, N, P	N, N, N	P, N, P	P, P, P	P, P, P
2-octenal	N, N, N	N, N, N	N, N, N	N, P, N	N, N, N	N, N, N	N, N, N	N, N, N
2-decen-1-ol	P, N, N	P, N, N	N, N, N	N, N, N	N, N, N	N, N, N	N, N, P	N, N, P
1-octanol	N, N, N	N, N, N	N, N, N	N, N, P	N, N, N	N, N, N	N, N, N	N, P, N
Limonene	N, N, N	N, N, P	N, N, N	N, N, N	N, N, N	N, N, N	N, N, N	N, N, P
D-limonene	N, N, N	P, P, N	P, P, N	P, P, P	N, N, N	N, N, N	N, N, N	N, N, N
Nonanal	P, P, P	P, P, P	P, P, P	P, P, P	P, P, P	P, P, P	P, P, P	P, P, P
Decanal	N, N, P	P, N, N	P, N, N	P, P, P	N, N, N	N, N, N	N, N, N	N, N, N

*N: Negative, P: Positive.

The data showed that the pH level was very effective in the formation of volatile compounds. Particularly, most of the volatiles significantly increased in the samples at pH 6.3 at later periods of storage. In each sample, hexanal formed and had the highest peak, as was expected. In addition to hexanal, the samples at both pH levels expressed heptanal, pentanal, 2,3-octanedione, 1-octen-3-ol, octanal, nonanal, and decanal.

**Figure 3:**
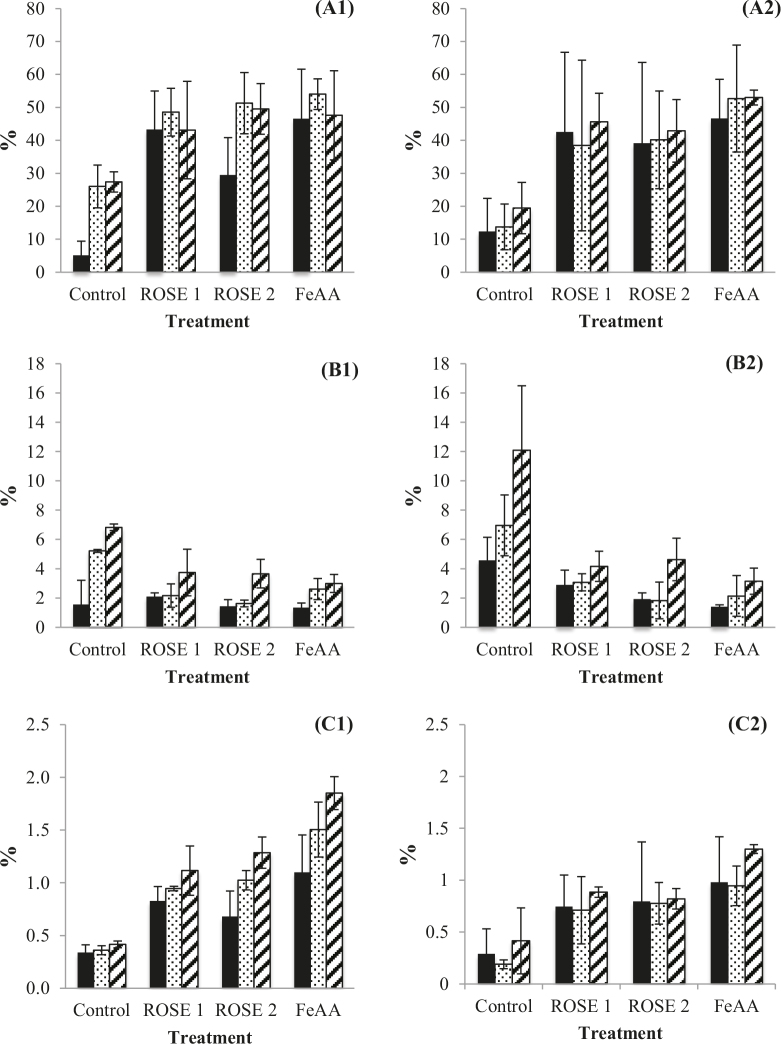
Hexanal (A), heptanal (B), and octanal (C) percentages of the WTMS samples at pH 6.3 on days 0 (solid), 3 (dotted), and 5 (stripped). (1) Samples treated at pH 6.3 and (2) samples treated at pH 6.8. Control: without oxidation initiator or ROSEs, FeAA: with oxidation initiator and FeAA, and ROSE 1 and ROSE 2 with oxidation initiator and ROSEs.

Figure 3 depicts the percentages of hexanal, heptanal, and octanal in relation to the lipid oxidation in the WTMS samples. Hexanal formation was associated with the linoleic acid oxidation of the farm-raised fish samples [35]. Hexanal is an important indicator for the determination of lipid oxidation [36]. Hexanal was in high amounts in each sample on each sampling day. This was an expected outcome, since hexanal is the main volatile responsible for rancidity in meats. Thus, heptanal can be directly related toflavor deterioration in fish and meat [28]. Figure 3 shows that the percentages of 3 volatile compounds were highly observed during storage of the samples. Hexanal formation was lower in both control samples than in the other tested samples (Figures 3A1 and 3A2) and ROSE-treated groups, and was similar at both pH levels. On day 3 of storage, the samples generally expressed the highest hexanal levels, except for the control groups. In the sample with FeAA at pH 6.3, it was significantly higher than in the other samples, but hexanal formation was observed at each sampling time, regardless of the treatment (P ≤ 0.05). Heptanal was measured at the highest levels in the control groups (Figures 3B1 and 3B2). Heptanal formed in similar trends in the treatment groups. The FeAA groups had significantly low heptanal levels on day 1 (P ≤ 0.05). Treatment with ferrous sulphate-ascorbic acid might have somehow suppressed the formation and/or release of heptanal.

The octanal comparison results are shown in Figures 3C1 and 3C2. Octanal formation was higher in the samples at pH 6.3, and generally increased during storage (P ≤ 0.05). The control groups had the lowest octanal levels at both pH levels. When the ROSE-treated groups were compared to the FeAA groups, the ROSEs relatively suppressed octanal formation in the samples at both pH levels.

### 3.4. Fatty acid profile

The total and individual FA profiles are shown in Table 2. Oleic acid (C18:1, n-9, c) and palmitic acid (C16:0) were found to be the most abundant unsaturated and saturated FAs at any time during storage, respectively. Other determined FAs were listed as palmitoleic acid (C16:1), stearic acid (C18:0), linolenic acid (C18:3, n-3), eicosadienoic acid (C20:2), behenic acid (C22:0), lignoceric acid (C24:0), and eicosapentaenoic acid (EPA, C20:5, n-3). On day 0, the samples at pH 6.3 had approximately 54% and 34% unsaturated and saturated FAs, respectively. The unsaturated and saturated FA levels were detected as 52% and 36%, respectively, in the samples at pH 6.8. On day 5, the percentages of FAsin the control groups did not change. in the samples at pH 6.3, approximately 2% decrease in the total unsaturated FAs and approximately 1.5% increase in the total saturated FAs were observed in the FeAA, ROSE 1, and ROSE 2 groups. The total unsaturated FAs decreased approximately 1% and total saturated FAs increased approximately 1% in the FeAA, ROSE 1, and ROSE 2 groups at pH 6.8. The decrease in unsaturated FAs might have occurredbecause the polyunsaturated FAs oxidize during the process of storage. The increase in saturated FAs might have been caused by the oxidation of unsaturated FAs. Since saturated FAs are stable against lipid oxidation, the increase in percentage might have been related to the decreased percentages of unsaturated FAs as a result of lipid oxidation. The FA profile of the study was found to be in accordance with the results of Thiansilakulet al. [3], who observed that the FA profile of red tilapia consisted of oleic acid (C18:1, n-9, c), followed by palmitic (C16:0) and linoleic acid (C18:2, n-6, c), for days 0 and 15 of iced storage. As the hydrolysis of triglycerides and phospholipids of red tilapia occurred, decreases in the docosahexaenoic acid and eicosapentaenoic acid (11.63% and 33.13%, respectively) were observed, which were caused by the high susceptibility of polyunsaturated FAs to lipid oxidation. Even the washing steps might have cause lipid oxidation. The pH level was another effective factor in the oxidation of polyunsaturated FAs. Significant decreases were obtained in the polyunsaturated FA levels of the samples prepared at pH 6.8 (P ≤ 0.05).

**Table 2:** Total saturated, monounsaturated, polyunsaturated, and unsaturated FA profiles of the samples taken on storage days 0 and 5.

		*Treatment (% mean ± SD)			
Total FAs	Time, pH	Control	ROSE 1	ROSE 2	FeAA
Saturated	Day 0, pH 6.3	34.3 ± 0.79bB	35.3 ± 0.54aC	35.8 ± 0.72aC	35.2 ± 0.48aC
	Day 5, pH 6.3	35.7 ± 0.30bB	36.3 ± 0.35abB	36.3 ± 0.06abBC	36.5 ± 0.45aB
	Day 0, pH 6.8	36.3 ± 0.13aB	36.1 ± 0.08aB	36.6 ± 0.44aAB	36.4 ± 0.12aB
	Day 5, pH 6.8	36.2 ± 0.08bA	37.0 ± 0.35aA	37.2 ± 0.13aA	37.4 ± 0.01aA
Monounsaturated	Day 0, pH 6.3	37.7 ± 0.36aA	36.1 ± 0.73bA	37.0 ± 0.77aA	37.9 ± 0.54aA
	Day 5, pH 6.3	36.4 ± 0.23aB	35.4 ± 0.24bA	35.5 ± 0.07abB	35.7 ± 0.23abB
	Day 0, pH 6.8	35.5 ± 0.31aB	35.3 ± 0.17abA	35.7 ± 0.59abB	34.5 ± 0.73bC
	Day 5, pH 6.8	35.9 ± 0.69aB	35.3 ± 0.23abA	34.8 ± 0.03bB	35.0 ± 0.51abBC
Polyunsaturated	Day 0, pH 6.3	16.3 ± 0.22aA	16.6 ± 0.10aA	16.2 ± 0.19aA	16.2 ± 0.01aA
	Day 5, pH 6.3	15.6 ± 0.19aB	15.4 ± 0.28aB	15.4 ± 0.18aB	15.2 ± 0.30aB
	Day 0, pH 6.8	16.6 ± 0.10aA	16.4 ± 0.28aA	16.5 ± 0.09aA	16.5 ± 0.07aA
	Day 5, pH 6.8	15.6 ± 0.02aB	15.1 ± 0.25bB	15.2 ± 0.33bB	15.2 ± 0.12bB
Unsaturated	Day 0, pH 6.3	54.1 ± 0.58aA	52.6 ± 0.63aA	53.2 ± 0.58aA	54.0 ± 0.55aA
	Day 5, pH 6.3	52.0 ± 0.04aB	50.8 ± 0.04bBC	50.9 ± 0.11bBC	50.9 ± 0.07bBC
	Day 0, pH 6.8	52.1 ± 0.41aB	51.7 ± 0.11abB	51.2 ± 0.66abB	51.0 ± 0.82bBC
	Day 5, pH 6.8	51.5 ± 0.39aB	50.4 ± 0.48bC	50.0 ± 0.30bC	50.2 ± 0.38bC

* Means with different lowercase letters in each row show significantly different treatment values (P ≤ 0.05). Means with different capital letters in each column show significantly different treatment values within each type of FA (P ≤ 0.05). Treatments are noted as the Control: without oxidation initiator or ROSEs, FeAA: with oxidation initiator and FeAA, and ROSE 1 and ROSE 2 with oxidation initiator and ROSEs.

This study showed the importance of the sample pH level in the case of using an external antioxidant to inhibit the occurring lipid oxidation in WTMS samples. Polyphenols extracted from ROSEs were more effective against the oxidation of lipids at a higher pH level. One of the main differences of this study from previous washed meat studies was that a ferrous sulfate-ascorbic acid complex was used as the oxidation initiator instead of a hemoglobin solution. In this study, the ferrous sulfate-ascorbic acid complex showed similar oxidation trends observed in previous studies prepared with hemoglobin. Moreover, the application of the ferrous sulfate-ascorbic acid complex had an additional advantage as preparation of the initiator solution with a minor deviation. Although onion skin is generally considered as waste, polyphenols extracted from the skin (or flesh) may be considered as a promising external antioxidant to suppress lipid oxidation. As future studies, the antioxidant effectiveness of vegetable and fruit extracts can be tested on different washed meat products and whole meat products.

## 4. Conclusion

In this study, the effects of pH and the addition of ROSEs on lipid oxidation of a WTMS were investigated. A ferrous sulfate-ascorbic acid complex was used to initiate lipid oxidation at pH levels of 6.3 and 6.8, because the pH of fish is usually measured as between 6 to 7.0 and tends to decrease as time passes. A WTMS at a lower pH level was believed to be more prone to lipid oxidation. As a result, higher amounts of unsaturated FAs oxidized in the samples at pH 6.3 than observed in the samples at pH 6.8. The addition of ROSE decreased the PV and TBARS levels when compared to the FeAA samples. Even lower amounts of ROSE showed promising inhibition on lipid oxidation. The onion skins could be an alternative antioxidant source and easily be applied to foods with high fat contents to extend lipid oxidation initiation, and thus, improve their shelf lives.
